# DNA Molecules as Standard Reference Materials I: Development of DNA Identification Sequences and Human Mitochondrial DNA Reference Sequences

**DOI:** 10.6028/jres.102.005

**Published:** 1997

**Authors:** Keith McKenney, Joel Hoskins, Jingxiang Tian, Prasad Reddy

**Affiliations:** National Institute of Standards and Technology, Gaithersburg, MD 20899-0001; Center for Advanced Research in Biotechnology, University of Maryland Biotechnology Institute, 9600 Gudelsky Drive, Rockville, MD 20850; National Institute of Standards and Technology, Gaithersburg, MD 20899-0001

**Keywords:** deoxyribonucleic acid (DNA) sequencing, DNA identification, genetic codes, genetic information, molecular tags, standard reference materials

## Abstract

This paper describes the design, construction and characterization of two DNA molecules that function as prototype Standard Reference Materials for use in the determination of DNA sequences. The first prototype reference DNA molecule was constructed to function as a true reference DNA sequence encoding the human mitochondrial hypervariable region 1. The second DNA molecule was designed to be a molecular DNA “tag” for use in the identification of DNA Standard Reference Materials. DNA molecules were chemically and enzymatically synthesized, cloned in molecular cloning vectors and sequenced using manual and automated fluorescent DNA sequencing methods. The sequence of the cloned human mitochondrial HV1 DNA shows six base pair differences from the original published DNA sequence. The molecular DNA tag sequence uses the universal genetic code with one letter amino acid abbreviations to spell the words “NIST DNA STANDARD REFERENCE MATERIAL.” The need and utility of an expanded molecular code to uniquely identify DNA molecules is discussed. The use of these DNA molecules in the development of DNA Standard Reference Materials is also described.

## 1. Introduction

The second half of this century has seen a remarkable scientific revolution in our capacity to elucidate, interpret and manipulate genetic information. This revolution was created by modern molecular biology and results from our ability to efficiently manipulate, purify, synthesize, sequence, and express DNA. These techniques provide the foundation for the modern multi-billion dollar biotechnology industry. DNA sequence determination of genes and genomes provides unprecedented access to the genetic structure of deadly viruses as well as life saving gene products.

The principle method used today for DNA sequence determination is called the “dideoxy” method and was developed by Fred Sanger and Alan Coulson in the 1970’s [1]. Most of the DNA sequences determined over the past 15 years have relied on the manual reading of an autoradiographic image of size fractionated radioactively labeled DNA sequencing reaction products and has required considerable skill to accurately deduce and assemble DNA sequences.

The development and commercialization of automated DNA sequencing instruments [2–7] has resulted in the wide spread use of “automated” DNA sequence determination hardware and software. The computer software “calling” the DNA sequence from data generated by the instruments removes a large part of the human interpretation of data. The quality and accuracy associated with the automated DNA sequence determination has been demonstrated in many cases to be as good or better than manual methods.

DNA sequence quality is quite variable and is influenced by many factors that contribute to the overall process of DNA sequence determination. These factors include “wetware,” hardware, and software. The wetware consists of the chemistry, enzymology, and molecular biology that produce the DNA substrates and DNA sequencing reaction products. An instrument’s hardware resolves and detects the reaction products and the software “calls” the DNA sequence from the complex collection of peaks produced by the hardware. High quality, well designed DNA molecules of precise sequence would be useful in optimizing and characterizing instrument performance as well as providing internal standards that can be used to measure and document DNA sequence quality.

DNA based standards have been developed over the past two decades to meet a wide variety of needs including molecular weight estimations of DNA, RNA and protein molecules [18]. The restriction fragment length polymorphism standard reference material (NIST SRM 2390) is the first NIST certified DNA standard. The second NIST certified DNA standard for PCR based human identification has recently been released (NIST SRM 2391). A number of new standards for DNA sequence determination are in development and prototype molecules have been assembled and characterized. Two examples of the prototype DNA sequencing standards are described in this paper.

Identification of biological samples and individuals has been revolutionized by the analysis of DNA sequences. Analysis of genomic DNA sequence has changed science and in particular medicine and forensic science in fundamental ways. When genomic DNA is limited or degraded by circumstance, the mitochondrial DNA is frequently analyzed for the purpose of human identification.

The human mitochondrial genome sequence has been determined [8] to consist of 16 569 base pairs of DNA and has a highly variable region called the “D loop” of approximately 680 base pairs (bp). The 400 bp hypervariable one (HV1) sub-sequence of the D loop has been used for human identification purposes. We have constructed and characterized a number of DNA clones of the D loop region. One of the clones containing the HV1 sequence from coordinates 15 975 to 16 420 [8] has been designated JT3 and is the first prototype DNA sequencing standard for the human mitochondrial HV1 region.

Ideally, NIST DNA standards should be chemically well defined, straightforward to construct and easily identified as molecules traceable to NIST. DNA sequencing standards represent an interesting example of self replicating molecules that can be easily propagated ad infinitum. This raises a number of interesting questions. One question is that of molecular identification, i.e., once the molecule is out of a labeled tube or bottle, how can it be identified? To address the question of identifying molecules at the molecular level and to provide one solution to this problem, a unique DNA sequence was designed that could be covalently integrated into any NIST DNA molecule to facilitate the identification of NIST DNA standards. A 108 base pair double stranded DNA molecule was synthesized that can be translated in one reading frame using the universal genetic code with the one letter amino acid abbreviation to “spell out” NIST *DNA *STANDARD *REFERENCE *MATERIAL. The * symbol represents a punctuation that is encoded by translation stop codons. This DNA sequence functions as a “molecular tag” to uniquely identify this DNA molecule and molecules derived from this parent molecule.

Nature has evolved the genetic code to specify twenty amino acids. This code with one letter amino acid abbreviations, allowed the construction of the molecular tag described in the preceding paragraph. It is useful and functional to uniquely identify NIST DNA materials. Because it contains only 20 letters, it cannot be used to adequately construct all molecular tag sequences that one might need. A description of alternative DNA encoded tags is presented in the discussion.

## 2. Materials and Methods

### Enzymes and chemicals

Restriction endonucleases, bacteriophage T4 DNA ligase, DNA polymerase, and polynucleotide kinase were purchased from New England Biolabs (Beverly, MA)^1^. Calf intestinal alkaline phosphatase was purchased from Boehringer-Mannhiem (Indianapolis, IN). Isopropyl-beta-D-thiogalactopyranoside, 5-bromo-4-chloro-3-indolyl-beta-D-galactoside, acrylamide, bisacrylamide, and phenol were obtained from Life Technologies Inc. (Gaithersburg, MD). Deoxynucleoside triphosphates and dideoxynucleoside triphosphates were purchased from Pharmacia LKB Biotechnology. 35S dATP was purchased from Du Pont-New England Nuclear.

### Bacterial Hosts and Plasmids

*Escherichia coli* TG1 was obtained from Dr. T. Gibson, Laboratory of Molecular Biology, Medical Research Council (Cambridge, England). M13mp18 (9) was obtained from New England Biolabs.

### DNA procedures

Single-stranded bacteriophage M13 DNA templates were isolated from the culture supernatants of TG1 infected with M13 phage grown in Luria-Bertani (LB) medium [10]. The replicative form of M13 DNA was purified as described by Messing [11]. Ligation of DNA fragments was performed as described by Maniatis et al. [12]. Human DNA was isolated from mouth scrapings with bucal swabs and used for isolation of mitochondrial HV1 sequences. PCR using synthetic primers (described below) was used for amplification of the HV1 region to produce a DNA fragment of 445 bp. This fragment was cloned into the SmaI site of M13mp18 in host TG1.

### DNA Synthesis and Purification

Oligonucleotides were synthesized with an Applied Biosystems model 380B DNA synthesizer. 5′ dimethoxytrityl bearing oligonucleotides were purified by reverse-phase HPLC on a Hamilton PRP-1 column (P/N 79426) using an increasing linear gradient of acetonitrile in 0.1 mol/L triethylammonium acetate, pH 7.0. The solutions containing HPLC purified oligonucleotides were concentrated to dryness in a vacuum centrifuge, dissolved in 200 μL of HPLC grade water, transferred to 1.7 mL microfuge tubes and again concentrated to dryness in a vacuum centrifuge. 5′ dimethoxytrityl groups were removed by dissolving the oligonucleotides in 30 μL of volume fraction of 80 % acetic acid in water and incubated at room temperature for 10 minutes. To remove the dimethoxytrityl alcohol, organic impurities and to convert the DNA to a sodium-salt form, oligonucleotides were ethanol precipitated by the addition of 5 μL of 3 mol/L sodium acetate, 100 μL of 100 % ethanol and incubated at 0 °C for 5 min and recovered by centrifugation in a microcentrifuge. Oligonucleotide pellets were washed with 500 μL of ice cold mass fraction of 80 % ethanol and trace amounts of ethanol removed in a vacuum centrifuge. The final oligonucleotide products were dissolved in HPLC grade water and quantitated by optical density measurement at 260 nm.

### Oligonucleotide sequences and assembly

Five oligonucleotides labeled NIST 1–5 with the sequences shown in Fig. 3 were synthesized and used to construct a 108 base pair double stranded DNA molecule encoding the NIST molecular tag. The purified oligonucleotides NIST 1–5 were phosphorylated with adenosine triphosphate (ATP) and polynucleotide kinase, annealed, ligated with bacteriophage M13mp18 SmaI treated recipient vector DNA [2], transformed into *Escherichia coli* strain TG-1, and plated on LB agar plates. M13 plaques were picked and phage DNA isolated as described [10]. Two oligonucleotides, (L strand: 5″-TTAACTCCACCATTAGCACC-3′, H strand: 5′-CACGGAGGATGGTGGTCAAG-3′) were synthesized, purified and used for amplification of the HV1 sequence.

### DNA sequence determination

DNA clones were sequenced using the M13 universal sequencing primer KMG2 (5′CACGACGTTGTAAAACGACGGCCAG3′). The DNA molecular tag sequence was determined manually using ^35^S dATP and Sequenase (United States Biochemicals, Cleveland, OH) by the dideoxy method of Sanger et al. [1] as modified by Biggin et al. [13]. Fluorescent DNA sequencing was done using an Applied Biosystems Division of Perkin-Elmer (ABD) (Foster City, CA) model 373.

The ABD DNA sequencing system uses four different fluorescent dyes to identify each base specific chain termination reaction. Each of the four bases is synthesized with a specific colored dye that emit at different wavelength maxima when excited by the argon laser light source. The four dideoxy reactions are pooled into a single sample and run together in one lane on a polyacrylamide gel. The fluorescent emission signature of each molecule as it passes a fixed point 24 cm from the origin is detected and recorded by an Apple Quadra 650 microcomputer using ABD software. Data analysis is carried out by the Applied Biosystems Analysis program and produces an “electropherogram” with the predicted DNA sequence above the data peak profiles.

## 3. Results

### 3.1 DNA Molecules With Human HV1 Sequences

**Fig. 1:**
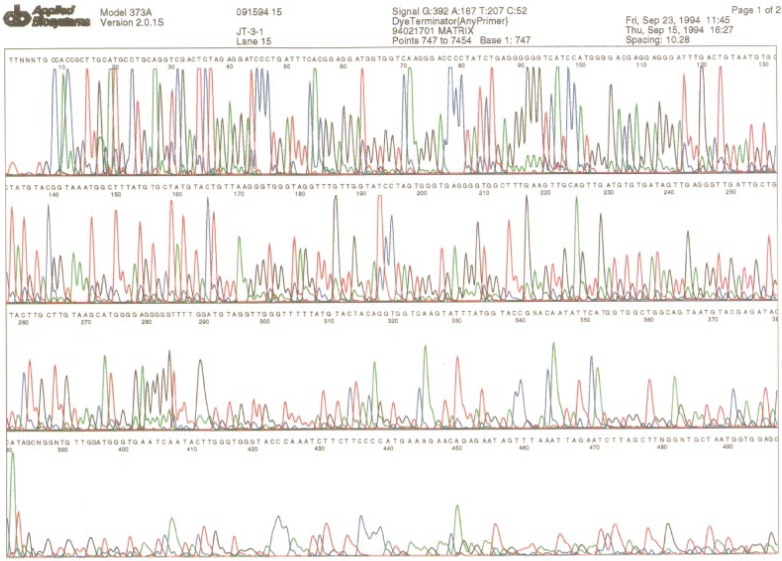
An electropherogram produced by an Applied Biosystems model 373A DNA sequencer from the JT3 DNA template using the universal KMG2 primer, dye terminators, and cycle sequencing as described in materials and methods. The peak data generated by the instrument was used by the instrument’s software to call the DNA sequence printed above each peak in the figure.

**Fig. 2:**
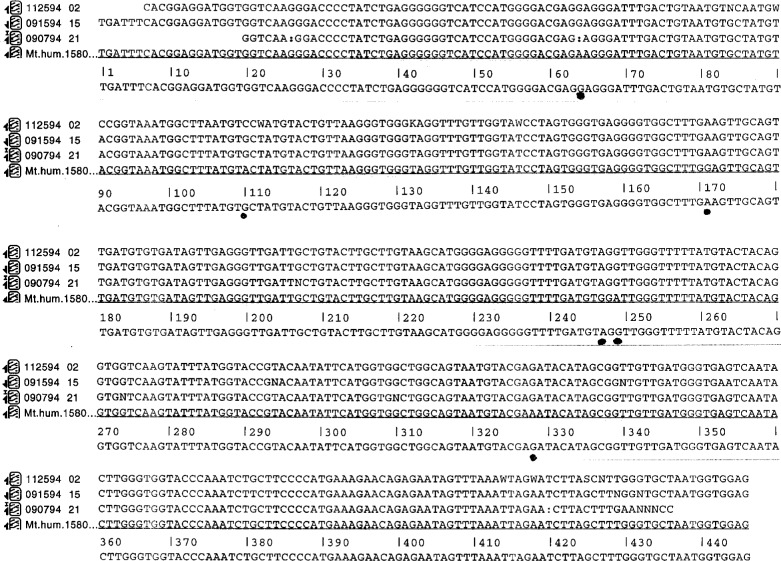
Three DNA sequences determined from: the HV1 PCR fragment cloned in M13 as clone JT3 (line 1), the HV1 PCR fragment sequenced directly using the H strand primer (the complement of this sequence is presented in Fig. 2 on line 3) and a clone of the HV1 PCR product in the opposite orientation of the JT3 clone (this sequence is presented in Fig. 2, line 11). These three sequences were used to generate a composite sequence of the HV1 fragment, shown in Fig. 2, line 12. The composite was compared with the Anderson DNA sequence (Fig. 2, line 14) [8] and showed six differences, denoted by the symbol *. No insertions or deletions were present in this HV1 sequence. A total of six bp differences are marked. See text for discussion.

Figure 1 presents an “electropherogram” of a cloned DNA fragment encoding human HV1 sequences produced by an Applied Biosystems Model 373 fluorescent based DNA sequencer. An electropherogram is an image constructed by the computer software that displays traces of peaks using four different colors each representing one of the four different dyes using in the DNA sequencing reaction. The peaks produced at the beginning of the electropherogram correspond to the smaller DNA fragments that reach the instrument’s detector first. Subsequent peaks correspond to DNA fragments of increasing length. The DNA template used to generate this data is an M13mp18 vector containing DNA sequences from the human mitochondrial HV1 region (from nucleotide 15975 to nucleotide 16420 [8] cloned into the SmaI site of the vector. The orientation of the HV1 insert relative to the polylinker sites of the M13mp18 vector is: 5′ EcoR1-SmaI-HV1 nucleotide 15975-nucleotide 16420-SmaI-HindIII-3′. This means the L strand sequence [8] corresponds to “top” strand of the M13 vector relative to the Plac promoter upstream of the M13 polylinker site. This DNA molecule has been designated JT3. The DNA sequence shown in Fig. 1 is the H strand sequence and was sequenced with the KMG2 universal primer using dye labeled dideoxy terminator chemistry with the Taq polymerase in a cycle sequencing reaction [17]. This sequence (091594 15) is also presented in figure 2, line 2.

The HV1 PCR product was sequenced directly using the H strand primer with dye terminator chemistry. This sequence (112594 02) is presented in Fig. 2 on line 1. In addition, the DNA sequence of a clone of the HV1 PCR product in the opposite orientation of the JT3 clone was is determined. This sequence (090794 21) is presented in Fig. 2, line 3. These three sequences were used to generate a composite sequence of the HV1 shown in Fig. 2, line 5. The composite was compared with the Anderson DNA sequence (Mt. hum. 1580 in Fig. 2, line 4) [8] and showed six differences. The six differences are marked with solid dots below the composite sequence.

Inspection of the data suggests that these differences are real and are representative of natural differences among individuals in this sequence. The differences were either A for G or G for A substitutions and are denoted with solid dots in Fig. 2. No insertions or deletions were observed. In addition, the differences were found in all three DNA sequences analyzed; the cloned HV1 PCR fragment in JT3, the HV1 PCR fragment cloned in the opposite orientation in M13 and the HV1 PCR fragment.

### 3.2 DNA Molecules With Tag Sequences

**Fig. 3:**
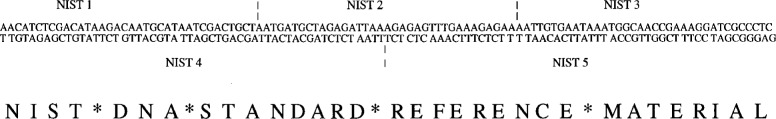
Nucleotide sequence of a synthetic double stranded DNA molecule designed to function as a molecular DNA tag. The upper strand has been translated using the universal genetic code with one letter abbreviations for the amino acids. The asterisks represent stop codons and are used to punctuate translated sequence.

Figure 3 presents the DNA sequence designed to encode the codons specifying the DNA molecular tag. The first triplet, AAC, encodes asparagine, which is abbreviated N. The second triplet ATC encodes isoleucine, which is abbreviated I. The third triplet, TCG, encodes serine, which is abbreviated S. The remaining base triplets encode the amino acid written below the sequence with the exception of TAA which encodes a stop codon, denoted by the * symbol in Fig. 3. This was used for punctuation of the molecular tag words. Note that subsequent asparagines are encoded by the triplet AAT. The double stranded DNA molecule was constructed from five single stranded synthetic oligonucleotides as described in materials and methods. The assembled double stranded sequence was cloned into the SmaI site of the M13 mp18 vector and clones with insert DNA were purified and characterized by DNA sequence analysis using two DNA sequencing methods.

**Fig. 4:**
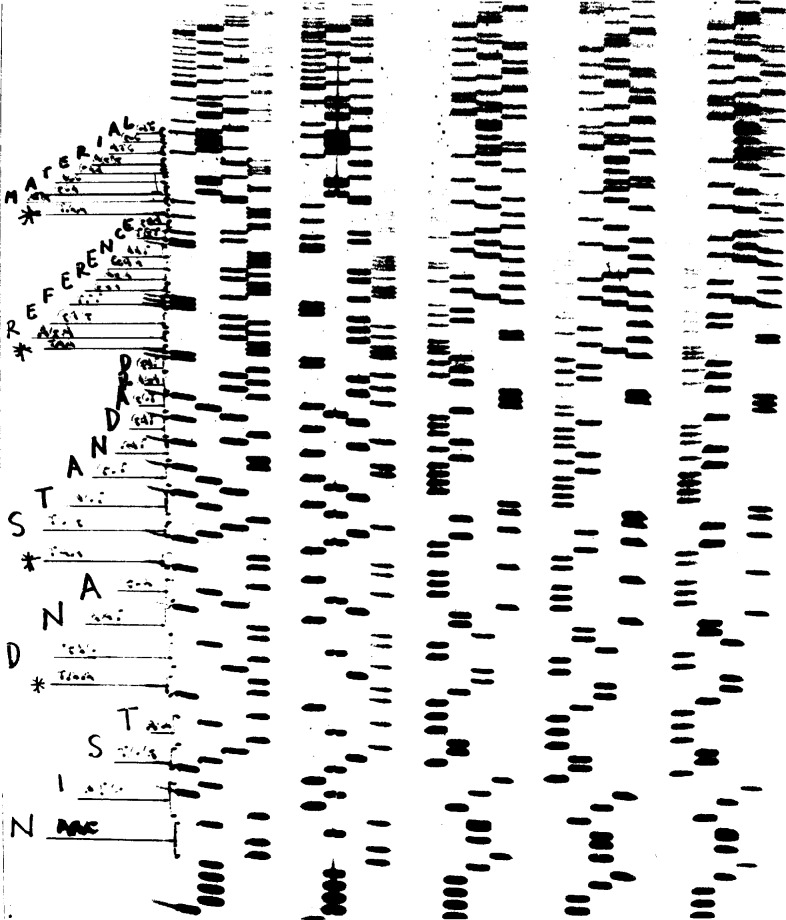
An autoradiographic image of a DNA sequencing gel. The DNA sequence is determined by “reading” the length and position of each DNA molecule. The DNA sequence written on the side of the x-ray film provides orientation and interpretation of the film image.

Figure 4 shows an autoradiograph of the sequence of five clones with the sequence of the left most clone (KM4) written in the margin. The sequence noted starts at the boundary between the M13 cloning vector’s SmaI site (5′-CCCC-3′) and the cloned insert DNA (5′-AACA-3′). The DNA sequence determination shows that all 108 bp are contained in this clone and are in the precise order shown in Fig. 3. No additional bp are apparent from this analysis.

**Fig. 5:**
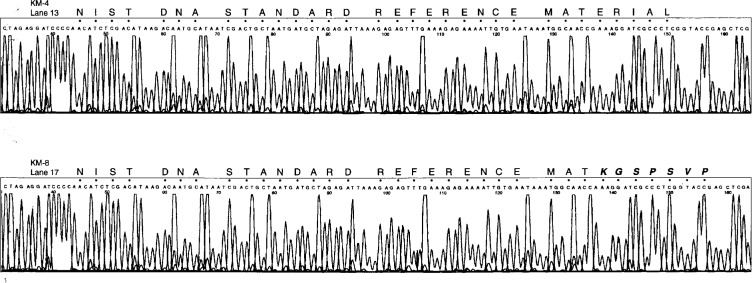
Parts of two electropherograms of the DNA sequencing products resolved and analyzed on an Applied Biosystems Model 373 DNA sequencer using protocols provided by the manufacturer. The upper electropherogram (positions 44 to 152) shows the 108 base sequence shown in figure one with the translation of the sequence written above the data. The lower electropherogram is missing a single base (at position 137, a G in the upper electropherogram) which results in a frame shift type mutation in the decoded tag sequence shown above the data.

Figure 5 (top part) shows the DNA sequence of clone KM4 using the ABD Model 373A automated DNA sequencing instrument with fluorescent dye terminator chemistry. The sequence noted above each peak in the electropherogram represents the automated DNA base calling software version 2.0.1 used on the Applied Biosystems 373A. The transition between the M13 cloning vector DNA sequence (5′-CCCC-3′) and the cloned insert DNA sequence (5′-AACA-3′) occurs between positions marked 43 and 44 in the figure. The insert DNA sequence continues to position 151 at which point the sequence returns to the vector sequence (at position 152). The 108 bp sequence is complete as determined using this method.

The DNA sequence determined by both methods is in agreement with the sequence represented in Fig. 3. Sequence of DNA clones with this insert in the opposite orientation (data not presented) are also consistent with the DNA sequence shown in Fig. 3. These three independent data sets demonstrate that the molecular DNA tag sequence has been accurately synthesized, cloned and can be accurately sequenced using standard manual and automated methods.

### 3.3 DNA Molecules With Tag Sequence Errors

Figure 5 (bottom half) shows the DNA sequence of clone KM8 using the fluorescent dye terminator chemistry on the 373A instrument. The DNA sequence data demonstrates that a single bp has been deleted in this clone, corresponding to the G at position 137 in clone KM-4 shown in the upper electropherogram. This single bp deletion produces a frame shift mutation in the molecular tag sequence written above the DNA sequence in the figure. The word “MATERIALS” is now altered to the nonsense word “MATNGSPSV”. The single bp deletion probably results from an error in either the synthesis and assembly of the oligonucleotides into double stranded DNA or during the cloning process.

## 4. Discussion

We have designed, constructed and characterized by DNA sequence analysis a number of DNA molecules for use as reference materials. Two of these molecules have been selected as prototype standard reference materials to facilitate DNA sequence determination.

DNA molecules that function as true reference sequences can be constructed to encode any one of a number of different sequences. We chose to construct a clone encoding the human mitochondrial HV1 sequence because of the widespread use of this sequence in the identification of humans and human tissues. The HV1 region is among the most variable in the mitochondrial genome, with one bp difference every one hundred nucleotides, on average [16]. The HV1 sequence was amplified by PCR, cloned in bacteriophage M13 and the sequences of both the amplified DNA as well as the cloned sequences were determined. The sequence shows that in fact, the JT3 clone that we have constructed contains six bp differences with the published sequence over a sequence of 445 bp, as shown in Fig. 2.

Reference molecules can be used as internal standards to measure and document the quality of DNA sequence determination. The JT3 molecule that contains the HV1 cloned fragment should prove useful to the forensic applications that analyze human mitochondrial DNA sequences [16]. In addition, the JT3 molecule provides a useful DNA template for interlaboratory testing of the overall DNA sequencing process.

One interesting problem presented by the use of recombinant DNA molecules of the M13 type described above for the human HV1 sequences is the problem of labeling or tagging the recombinant molecule as a NIST reference material. One solution to the tagging of DNA molecules is to construct a DNA sequence that can spell words that are easily recognized.

A number of DNA molecular tag sequences were considered. Three components were considered important in the design. The first component was ease of identification and connecting the sequence to the National Institute of Standards and Technology. This was accomplished by creating a sequence that could be translated using the universal genetic code into protein sequence with the one letter abbreviation used for each amino acid. In addition, the DNA sequence coding for the translation stop codon (TAA) was used to separate and punctuate each word. The second design component important in the tag sequence was size of the DNA sequence. The DNA sequence needed to be large enough to be easily identified but small enough to be completely sequenced in one “pass” through the sequence using current DNA sequencing methods. The current limit of “one pass” sequencing is about 450 bases of sequence data. This limit is increasing because of longer gels, better DNA polymerases and improved software and should routinely approach 600 to 700 bases over the next several years. The third component was the simplicity of the sequence such that it could be synthesized, cloned and accurately sequenced.

We designed a 108 bp DNA sequence that satisfies the three design components by using the universal genetic code with one letter amino acid abbreviations to spell the words “NIST DNA STANDARD REFERENCE MATERIAL” shown in Fig. 3. The DNA sequence has a fairly random distribution of bases with no obvious secondary structural components. Five oligonucleotides were synthesized based on the sequence shown in Fig. 3 as described in materials and methods. The oligonucleotides were assembled, cloned in M13mp18 and characterized by manual and automated DNA sequence analysis.

The data presented in Fig. 4 was generated using the manual dideoxy method with the Sequenase DNA polymerase. The data presented in Fig. 5 was generated using the dye terminator method with the Taq DNA polymerase in a cycle sequencing format on an ABI 373. Both methods show that the precise 108 bp sequence shown in Fig. 3 is present in the clone designated KM4. Characterization of an additional clone, KM8 shown in Fig. 5 (lower half), demonstrated that a single bp (the G at 137 in KM4) has been deleted in this clone. The single bp deletion causes a frame shift mutation and produces an altered non-sense molecular tag sequence, MATNGSPSV instead of MATERIALS, as shown in Fig. 5.

The molecular tag sequence used in this work was constructed using the twenty letters of the naturally occurring amino acids specified by the “universal” genetic code. This scheme has six letters missing from the English alphabet and has no numbers or punctuation other than stop codons which limit its utility in designing and constructing molecular tags. One could create a triplet code similar to the universal code that Nature evolved and eliminate the code degeneracy. An example of such a simple code would include all 26 letters of the English alphabet, the numbers 0 through 9 as well as commonly used punctuation characters. Another alternative code would be a tetranucleotide code using all four DNA bases to provide 256 unique codons. One could use 255 codons to specify the complete set of characters in the ISO Latin-1 character set. These codes could be used to design and synthesize DNA oligonucleotides that could be assembled into mini-genes and used by any organization to identify their products using molecular tags in conjunction with DNA sequence determination. Both of these coding schemes have the advantage of being easily recognized because that words are spelled in English and no central database is required to maintain the code. A more efficient code would be to eliminate the need to spell words and simply use a digital code in the base four. Code lengths of 30 bp could provide 4^30^ possible tags. Such a coding scheme would require a standard form and databases as well.

The ability to efficiently synthesize DNA oligomers in milli-molar quantities exists as well as the ability to detect just a few DNA molecules using the polymerase chain reaction. This provides the requisite technologies for a universal tagging scheme to identify many biological and nonbiological materials.

The DNA sequences described in this paper represent the first generation of standard reference DNA molecules that are designed, synthesized, constructed and demonstrated to have precise, well defined chemical composition and molecular properties. The NIST tagged DNA molecules provide the materials for constructing the next generations of DNA standard reference materials.

A second generation of standard reference DNA molecules is under construction and has been designed with complex DNA sequences that function as test substrates for the different components in DNA sequence analysis. These components include DNA replication, enzymology, electrophoretic fragment separation, detection, and software analysis.

In summary, this paper presents and describes two new DNA molecules for use as Standard Reference Materials that are *designed* to have specific chemical and biochemical properties and then *synthesized, cloned, and characterized* to have the correct DNA sequence.

## Footnotes

1 Certain commercial equipment, instruments, or materials are identified in this paper to foster understanding. Such identification does not imply recommendation or endorsement by the National Institute of Standards and Technology, nor does it imply that the materials or equipment identified are necessarily the best available for the purpose.
